# Virtual Sensor for Kinematic Estimation of Flexible Links in Parallel Robots

**DOI:** 10.3390/s17091934

**Published:** 2017-08-23

**Authors:** Pablo Bengoa, Asier Zubizarreta, Itziar Cabanes, Aitziber Mancisidor, Charles Pinto, Sara Mata

**Affiliations:** 1 Department of Automatic Control and System Engineering, Faculty of Engineering in Bilbao, University of the Basque Country (UPV/EHU), Plaza Ingeniero Torres Quevedo 1, 48013 Bilbao, Spain; pablo.bengoa@ehu.eus (P.B.); itziar.cabanes@ehu.eus (I.C.); aitziber.mancisidor@ehu.eus (A.M.); sara.matac@ehu.eus (S.M.); 2 Department of Mechanical Engineering, Faculty of Engineering in Bilbao, University of the Basque Country (UPV/EHU), Plaza Ingeniero Torres Quevedo 1, 48013 Bilbao, Spain; charles.pinto@ehu.eus

**Keywords:** flexible link manipulator, parallel robots, kinematics, Finite Element Method, virtual sensor

## Abstract

The control of flexible link parallel manipulators is still an open area of research, endpoint trajectory tracking being one of the main challenges in this type of robot. The flexibility and deformations of the limbs make the estimation of the Tool Centre Point (TCP) position a challenging one. Authors have proposed different approaches to estimate this deformation and deduce the location of the TCP. However, most of these approaches require expensive measurement systems or the use of high computational cost integration methods. This work presents a novel approach based on a virtual sensor which can not only precisely estimate the deformation of the flexible links in control applications (less than 2% error), but also its derivatives (less than 6% error in velocity and 13% error in acceleration) according to simulation results. The validity of the proposed Virtual Sensor is tested in a Delta Robot, where the position of the TCP is estimated based on the Virtual Sensor measurements with less than a 0.03% of error in comparison with the flexible approach developed in ADAMS Multibody Software.

## 1. Introduction

Since their introduction in the industry back in the early 1960s, robots have been considered as a cornerstone of the mass-production system due to their capacity to combine minimal cost with adaptability, quality and high productivity. Although automotive industry has been the predominant user, in the last decade there has been a growing interest on advanced robot technology in other areas such as food, pharmaceutics or manufacturing industries.

In the current global market, smaller production time and higher quality products are required to be competitive. Robotic applications in industry need to be fast and accurate enough to satisfy these requirements. However, in serial robots, which have been traditionally used in industry, the increase in speed usually implies a loss in accuracy. Hence, when both requirements need to be met, the use of Parallel Kinematic Robots (PKR) [1] has been proposed. These robots are composed by two platforms connected by multiple kinematic chains, being one of them fixed (base platform), and the other, where the Tool Centre Point (TCP) is located, mobile.

Parallel robots have a series of advantages in comparison with their serial counterparts, such as higher load/weight ratio, and higher stiffness and precision. Furthermore, since the motors can be fixed to the base platform, the moving mass is reduced and higher acceleration and speed can be achieved. These characteristics improve the performance of the robot in terms of working efficiency, precision and energy consumption [2].

To address the need for achieving higher productivity, manufacturers have tried to reduce the moving mass of the robots by decreasing the cross-section of the limb. This, in addition to the high accelerations required for low operation cycles, results in a certain degree of elastic deformation in the limbs [2]. These deformations can cause substantial errors in motion control due to their influence in both dynamics and kinematics of these robots. In fact, one of the most difficult problems caused by elastic deformation is TCP trajectory tracking, as deformations need to be compensated to maintain the required accuracy.

In stiff or undeformable robots, the location of the TCP can be determined using the active joint position sensors, this is, the sensors attached to the actuators, and the kinematic model derived from its geometrical structure. However, in the case of flexible robots, the TCP location depends not only on joint positions but also on the deformation of its flexible links. Therefore, the stiff element assumption does not provide enough accuracy, as the deformation of the different flexible elements must be considered [3].

Control of flexible link manipulators, even single link manipulators, is a challenging task as these robots are of non-minimum phase [4]. When multiple links are considered, this problem increases in complexity due to the distributed flexibility. In addition, feedback controllers require the measurement or estimation of both joint motions and the flexible link deformation to guarantee accuracy and trajectory tracking while reducing the effect of vibrations caused by flexibility [4]. For this purpose, both rigid and flexible variables must be measured.

The measurement of deformations due to link flexibility has been carried out considering different types of sensors [5]. One of the approaches that provides better deformation data is the use of artificial vision, which has been used to determine the location of the TCP of flexible manipulators [6,7,8] and provide their calibration [9,10]. Vision systems can be used to implement visual servoing [11], that controls directly the TCP’s position of the robot without the use of estimators. Most of visual servoing approaches require the calculation of the image Jacobian and robot Jacobian to map end-effector velocity to image feature velocity [12]. In flexible manipulators, however, these Jacobians require the information of the flexible variables [13], which are still needed to be measured [14]. Hence, the measurement of the deformation of each link by the use of artificial vision systems has also been proposed [6,7], though this approach requires a camera per flexible link, increasing the cost of the robot. Hence, although precise and global data can be derived from vision systems, their use is limited by their view range, the effects of visual obstruction, link interference, low sampling rate and the computational cost required to process the images.

To overcome the limitations of vision systems, conventional sensors such as accelerometers or strain gauges are widely used. The accelerometers are usually placed at the tip of each flexible link, and their measurements are traditionally used to implement vibration control approaches in order to stabilize the oscillation of the TCP due to flexibility [15]. Hence, as tip accelerations contain both information of rigid-body motions and flexible-link vibrations, it is possible to estimate the TCP location of the manipulator if joint motions are sensorized [16]. This approach, however, provides noisy measurements, contains biases, and requires to integrate the acceleration signal twice for velocity and position estimations, resulting in high accumulation of errors [6].

On the other hand, strain gauges have been widely used to measure the local deformation of flexible links [16]. For instance, in [17] strain gauges were used in a three degree-of-freedom flexible robot links for control proposes. In [18] an interpolation algorithm to determine the tip position and orientation of a flexible beam is defined from a finite set of strain measurements. Finally, in [14] the bending and torsional deformations kinematic relations of a 3D flexible-beam were verified using strain measurements.

Strain gauges require only the knowledge of geometrical parameters of the link to estimate the deformation, without considering dynamic parameters such as link masses or inertias. Hence, they have become an attractive approach. However, strain gauges are prone to temperature variations and they inherently suffer from measurement noise [19] and biasing due to electromagnetic interferences [6,7].

Thus, the measurement of the deformation of flexible links is not a trivial task even with the use of additional sensors. Based on this fact, the use of sensor fusion techniques have also been proposed to improve the measurement: In [20] the fusion of accelerometer and encoder signals using a disturbance observer to compensate the nonlinearities of the deformation was proposed, while in [21], an extended Kalman filter to process accelerometer and encoder data was developed to estimate the forward kinematics of a 6 DOF robot. However, although the use of sensor fusion techniques improves the accuracy of the measurement, the proposed techniques usually require high computational load [22].

This work presents a novel Virtual Sensor that allows to estimate the deformation of flexible links based on the use of a single high resolution optical encoder and a mathematical model. This deformation is critical to accurately estimate the position and orientation of the TCP of flexible parallel robots. The proposed approach allows to calculate the deformation of the flexible links with minimal computational cost, providing significant advantages for Real-Time implementation over the aforementioned approaches.

The rest of this paper is organized as follows: Section 2 outlines concept and theoretical development of the presented virtual sensor. In Section 3, the theoretical development is applied into a Delta robot. The simulation results are discussed in Section 4. Finally, the most important ideas are summarized in the final section.

## 2. Theoretical Development of the Kinematic Virtual Sensor

**Figure 1:**
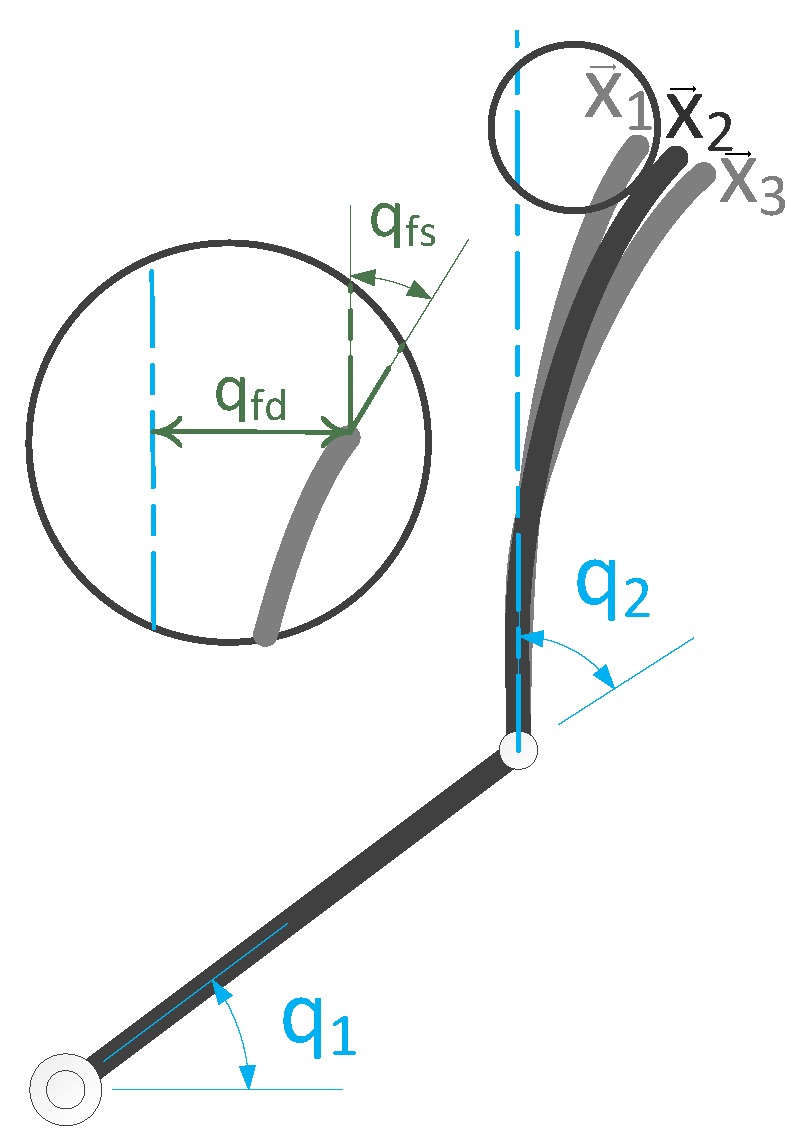
A flexible manipulator with the same configuration of rigid joints $q_{r}\left(q_{1},q_{2}\right)$ but multiple end-effector position/orientation due to the flexibility of the links, $q_{f}\left(q_{f_{d}},q_{f_{s}}\right)$. ($q_{f_{s}}$ link tip transverse flexural deflection and $q_{f_{s}}$ link tip flexural slope).

The Direct Kinematic Model of parallel robots allows to calculate the trajectory in the operational space x(t) for any given joint space trajectory q(t), this is, f(q(t))=x(t). Therefore, it is easy to see that for any flexible link manipulator, the direct kinematic equation admits multiple solutions, as the deformation of the link provides more than one end-effector position/orientation for the same configuration of joints q [23] (Figure 1).

Therefore, the Direct Kinematic Model, which is mandatory for robot control applications, has to be defined in terms of the rigid joint motion variables $q_{r}$ and the flexible variables $q_{f}$ that model the deformation of the flexible links,

(1) $$x\left(t\right)=f\left(q_{r},q_{f}\right)$$

where the flexible variables $q_{f}\left(q_{f_{d}},q_{f_{s}}\right)$ are composed by the link tip transverse flexural deflection $q_{f_{s}}$ and the link tip flexural slope $q_{f_{s}}$.

However, the number of degrees of freedom (DOF) of the system increases, and in order to estimate correctly the TCP of the robot, measurements of both rigid and flexible variables are required. Measurement of rigid joints can be easily carried out using extra sensors attached to rotary or linear joints. However, as stated in the introduction, the measurement of the flexible variables can be a challenging tasks. In this section, a novel Virtual Sensor based on a normal mode analysis approach is proposed that allows to estimate the deformation with enough accuracy and minimal computational cost.

### 2.1. Fundamentals of the Virtual Sensor

The normal mode analysis is a mathematical tool which represents a pattern of motion in which all parts of a system move with the same frequency and fixed phase relation. Hence, this mathematical tool provides the kinematic relation between the different DOF of the system based on its dynamic properties, such as their material, structure and boundary conditions. Furthermore, the general motion of a system is composed as a superposition of its normal modes, being all normal modes orthogonal to each others, since the excitation of one mode will not affect to a different mode. This way, if a particular mode is considered and the modal motion of a single degree of freedom measured, the complete set of DOF $q_{\mathrm{DOF}}$ modal motions can be estimated,

(2) $$q_{\mathrm{DOF}}=\sum_{k=1}^{n_{DOF}}X_{k}\;\epsilon_{k}$$

where $X_{k}$ is the *k*th eigenvector or mode shape and $\epsilon_{k}$ is the *k*th modal motion [24].

Several researchers [25,26,27] have suggested considering only the first few modes in the model neglecting high frequency since the amplitude terms related to them are much smaller. In addition, due to the damping properties of the materials, high frequency vibrations are softened more quickly than those of lower frequency [28]. On the other hand, in control applications of industrial manipulators, the bandwidth of the working frequencies is limited by the actuator system and the application itself. This, in addition to the use of rigid materials in the construction of robots, usually ensure that the second resonant frequency of the manipulator is out of the working bandwidth in all robot workspace. Hence, the deflection of the limbs can be estimated considering the mode associated to the first natural frequency of the manipulator. This way, Equation (2) can be simplified to,

(3) $$q_{\mathrm{DOF}}=\sum_{k=1}^{n_{DOF}}X_{k}\;\epsilon_{k}\approx X_{1}\;\epsilon_{1}$$

This constitutes the basis of the proposed Virtual Sensor. Let us assume that a robot presents a series of flexible links i=1,…,n connected with several stiff links. If the modal analysis of each link is carried out and the previous facts considered, the final deflection of the *i*th link can be estimated with a properly placed sensor that measures a single flexible DOF and the use of the model obtained after carrying out the modal analysis. Furthermore, if only the link is considered, the relation that is obtained in the modal analysis is constant for a given mode, resulting in a low computational cost approach for the estimation of the deflection of the link.

**Figure 2:**
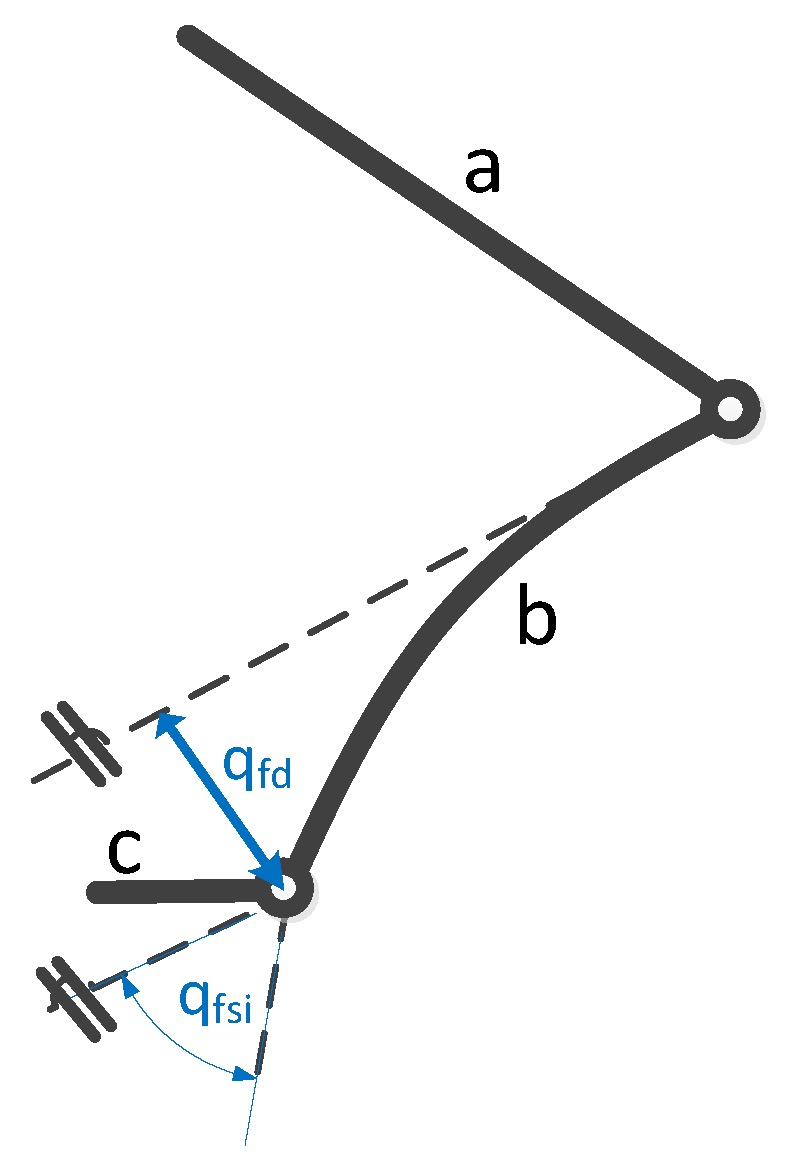
Schematic of a flexible link (*b*) connected with two stiff links (*a*, *c*) using rotatory joint.

Consider, for instance, the example of Figure 2, where the flexible link *b* is connected with two stiff links (*a*, *c*) using rotary joints. If Finite Element Method approach and Euler-Bernouilli Beam Theory is used to model the flexible link, one of the flexible variables, $q_{fs_{i}}$, would be the deformation slope at the tip of the flexible link. If a single high resolution optical encoder is used to measure this angular deformation and its data is introduced in Equation (3), by substituting $\epsilon_{1}=q_{fs_{i}}$, the total deflection at the tip of the link could be estimated, which is required to compute the TCP estimation of the robot.

This approach presents several advantages over previous approaches. First, as only the link is considered, the relation that is obtained in the modal analysis is constant for a given mode, resulting in a low computational cost approach for the estimation of the deflection of the link. Second, if small deflections are assumed, the approach provides the required accuracy for control purposes. Finally, since vector $X_{k}$ defines the relationship between the *k*th mode of the motion of the flexible DOFs of the *i*th link, the relationship holds not only for the position problem, but also for velocity and acceleration problems.

This way, full kinematic relations between the flexible variables,

(4) $$\begin{array}{c} q_{f_{i}}=\sum_{k=1}^{n_{DOF}}X_{k_{i}}\;\epsilon_{k_{i}}\approx X_{1_{i}}\;\epsilon_{1_{i}}\;{\dot{q}}_{f_{i}}=\sum_{k=1}^{n_{DOF}}X_{k_{i}}\;{\dot{\epsilon }}_{k_{i}}\approx X_{1_{i}}\;{\dot{\epsilon }}_{1_{i}}\\ {\ddot{q}}_{f_{i}}=\sum_{k=1}^{n_{DOF}}X_{k_{i}}\;{\ddot{\epsilon }}_{k_{i}}\approx X_{1_{i}}\;{\ddot{\epsilon }}_{1_{i}} \end{array}$$

Note, however, that the whole manipulator is not considered in the modal analysis, as it is highly dependant on the particular configuration of the robot. The consideration of the whole robot requires recalculation the modal analysis, and thus, high computational cost. Hence, the proposed approach presents a simplification of the procedure by considering only the flexible joints, $q_{DOF}=q_{f}$, allowing high computational efficiency while maintaining the required accuracy. In this work, the normal modes of each link are independent of the manipulator configuration, and only depend on the mechanical properties of the limbs and the discretization carried out based on the Finite Element Method approach. This allows to calculate the required matrices off-line, reducing the time to compute link deformation.

In the following section, the mathematical development of the presented virtual sensor is widely detailed.

### 2.2. Modelling of Flexible Links

In order to carry out the modal analysis of each flexible link and calculate their eigenvalues, the generalised inertia matrix and the stiffness matrix of each link has to be calculated. In this section, the procedure to calculate these matrices is detailed.

Finite Element Method (FEM) [29,30,31] and Assumed Modes Method (AMM) [32,33,34] approaches are the most used ones when modelling flexible links robots for control purposes. As the requirements and procedures of both approaches are different, several studies have been carried out to compare them [28,35,36]. This way, when computational cost is to be analysed, the aforementioned studies determine that FEM is a better approach due to its fewer computation requirements.

The Finite Element Method considers each flexible link *i* as an assemblage of a finite number, $n_{i}$, of small elements of length $l_{i}$ which are interconnected at certain points called nodes. Each element is referenced as ij, where subscript *j* denotes the number of the element. As it is well known, the more number of elements per link (hence, smaller elements), the more precise the overall solution of the system will be, making it converge to the exact solution as precisely as desired at the cost of higher computational cost.

**Figure 3:**
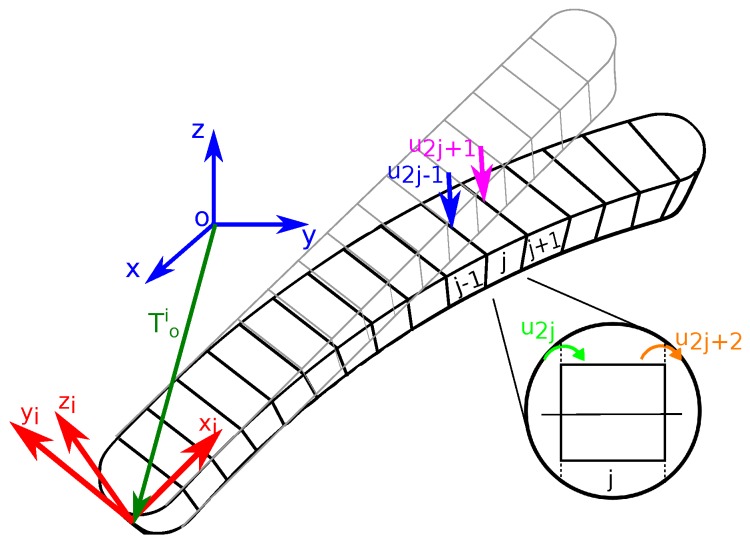
Schematics of the DOF of each link’s element.

In order to solve the dynamic problem, the Euler-Lagrange method is used, in which the energy of each element is to be analysed. However, before calculating the kinematic and potential energy, the position vector $r_{i}^{0}$, in inertial coordinates, of each element must be defined (Figure 3). For simplicity, this vector will be expressed in terms of a local coordinate vector $r_{i}$ [3]. This is accomplished by using the transformation matrix $T_{0}^{i}$, which relates the location of the reference system attached to each link $0_{i}X_{i}Y_{i}Z_{i}$ and the inertial system OXYZ,

(5) $$r_{i}^{0}=T_{0}^{1}\;\left[\begin{array}{c} L_{1}\\ 0\\ u_{{2n}_{1}+1} \end{array}\right]+T_{0}^{2}\;\left[\begin{array}{c} L_{2}\\ 0\\ u_{{2n}_{2}+1} \end{array}\right]+\ldots +T_{0}^{i}\;r_{i}$$

where

(6) $$r_{i}=\left[\begin{array}{c} \left(j-1\right)\;l_{i}+x_{ij}\\ 0\\ z_{ij} \end{array}\right]$$

According to Euler-Bernoulli beam theory, all elements are considered to possess two degrees of freedom (DOF) at each end of the element: a transverse flexural deflection ($u_{2j-1}$ and $u_{2j+1}$) and a flexural slope ($u_{2j}$ and $u_{2j+2}$) (Figure 3). These flexible DOF are related by the use of the shape functions $\phi_{k}\left(x\right)$, which describe the flexural displacement *z*, as

(7) $$z\left(x,t\right)=\sum_{k=1}^{4}\phi_{k}\left(x\right)\;u_{2j-2+k}\left(t\right)$$

where, for element *j* of the *i*th link the shape functions are defined by the following Hermitian polynomials [37,38],

(8) $$\begin{array}{cc} \phi_{1}\left(x\right) & =1-3\;\frac{x^{2}}{l_{ij}^{2}}+2\;\frac{x^{3}}{l_{ij}^{3}},\;\phi_{2}\left(x\right)=x-2\;\frac{x^{2}}{l_{ij}}+2\;\frac{x^{3}}{l_{ij}^{2}}\\ \phi_{3}\left(x\right) & =3\;\frac{x^{2}}{l_{ij}^{2}}-2\;\frac{x^{3}}{l_{ij}^{3}},\;\phi_{4}\left(x\right)=-\;\frac{x^{2}}{l_{ij}}+2\;\frac{x^{3}}{l_{ij}^{2}} \end{array}$$

where $l_{ij}$ is the length of the element and the displacement variables *x* and z(x) satisfy the following boundary conditions,

(9) $$z\left(0\right)=u_{2j-1},\;\frac{\partial z(0)}{\partial x}=u_{2j},\;z\left(l\right)=u_{2j+1},\;\frac{\partial z(l)}{\partial x}=u_{2j+2}\;$$

Once the position vector is defined, the kinetic energy $T_{ij}$ and potential energy $V_{ij}$ of each element are computed in terms of the generalised variables of the system $q={\left(q_{1},q_{2},\ldots ,q_{n}\right)}^{T}$ and their time derivatives $\dot{q}$ [39]. These energies are then summed to obtain the total kinetic *T* and potential *V* energies for the entire system. That is,

(10) $$T\left(q,\dot{q}\right)=\sum_{i=1}^{m}\sum_{j=1}^{n_{i}}T_{ij}$$

and

(11) $$V\left(q\right)=\sum_{i=1}^{m}\sum_{j=1}^{n_{i}}V_{ij}$$

where *m* is the number of links in the system and the kinetic energy $T_{ij}$ is obtained by integrating over the ij element’s length, $l_{ij}$, the corresponding energy function. Thus,

(12) $$T_{ij}=\frac{1}{2}\;\int_{0}^{l_{ij}}\;\rho_{i}\;A_{i}\;\left[\frac{\partial r^{T}}{\partial t}\;\frac{\partial r}{\partial t}\right]dx$$

which can be rewritten as,

(13) $$T_{ij}=\frac{1}{2}\;{\dot{z}}_{j}^{T}\;M_{ij}\;{\dot{z}}_{j}$$

where matrix $M_{ij}$ is the generalised inertia matrix of the element ij. This matrix is symmetrical and each (k,o) element of it is defined as,

(14) $$M_{ij}\left(k,o\right)=\int_{0}^{l_{i}}\;\rho_{i}\;A_{i}\;{\left[\frac{\partial r}{\partial z_{jk}}\right]}^{T}\left[\frac{\partial r}{\partial z_{jk}}\right]\;dx_{ij},\;o,k=1,2,\ldots ,n_{q}$$

where $\rho_{i}$ is the mass density, $A_{i}$ is the cross-section area of the element, $z_{jk}$ is the *k*th element of $z_{j}={\left[q_{r},u_{2j-1},u_{2j},u_{2j+1},u_{2j+2}\right]}^{T}$, $n_{q}$ is the number of the variables of the system and $q_{r}=\left[q_{1},q_{2},\ldots ,q_{n_{r}}\right]$ is the vector of variables associated to the stiff model.

The resulting generalized inertia matrix $M_{ij}$ with respect to a single rotation, $q_{r}=\left[q_{1}\right]$, is always defined as [3]:

(15) $$M_{ij}=\left[\begin{array}{cc} M_{ij}\left(1,1\right) & M_{ij}\left(1,2\right)\;M_{ij}\left(1,3\right)\;M_{ij}\left(1,4\right)\;M_{ij}\left(1,5\right)\\ M_{ij}\left(1,2\right) & \\ \vdots & P_{ij}\\ M_{ij}\left(1,5\right) & \end{array}\right]$$

where

(16) $$P_{ij}=\frac{m_{ij}\;l_{ij}}{420}\;\left[\begin{array}{cccc} 156 & 22l_{ij} & 54 & -13l_{ij}\\ 22l_{ij} & {4l}_{ij}^{2} & 13l_{ij} & -3l_{ij}^{2}\\ 54 & 13l_{ij} & 156 & -22l_{ij}\\ -13l_{ij} & -3l_{ij}^{2} & -22l_{ij} & 4l_{ij}^{2} \end{array}\right]$$

and,

(17) $$\begin{array}{ccc} M_{ij}\left(1,1\right) & = & \frac{\rho_{ij}\;A_{ij}\;l_{ij}^{3}}{3}\;\left(3j^{2}-3j+1\right)+\psi_{ij}^{T}\;P_{ij}\;\psi_{ij},\\ M_{ij}\left(1,2\right) & = & \frac{\rho_{ij}\;A_{ij}\;l_{ij}^{2}}{20}\;\left(10j-7\right),\\ M_{ij}\left(1,3\right) & = & \frac{\rho_{ij}\;A_{ij}\;l_{ij}^{3}}{60}\;\left(5j-3\right),\\ M_{ij}\left(1,4\right) & = & \frac{\rho_{ij}\;A_{ij}\;l_{ij}^{2}}{20}\;\left(10j-3\right),\\ M_{ij}\left(1,5\right) & = & \frac{\rho_{ij}\;A_{ij}\;l_{ij}^{3}}{60}\;\left(5j-2\right). \end{array}$$

with,

(18) $$\psi_{ij}={\left[u_{2j-1},u_{2j},u_{2j+1},u_{2j+2}\right]}^{T}$$

where $\rho_{ij}$ is the mass density, $A_{ij}$ the cross-section area and $l_{ij}$ the length of the $j^{th}$ element of the link *i*.

In the same way, by computing the potential energy for each element $V_{ij}$ of the link and adding their contributions, the overall potential energy of the system is obtained, Equation (11). However, unlike the kinetic energy, the potential energy is divided into two components: the potential energy due to the gravity ($V_{g_{ij}}$) and the potential energy due to the elasticity ($V_{e_{ij}}$). This is,

(19) $$V_{ij}=V_{g_{ij}}+V_{e_{ij}}=\int_{0}^{l_{ij}}\rho_{ij}\;A_{ij}\;g\;\left[0\;\;0\;\;1\right]\;r\;dx_{ij}+\frac{1}{2}\;\int_{0}^{l_{ij}}E\;I_{i}{\left[\frac{\partial^{2}y_{ij}}{\partial x_{ij}^{2}}\right]}^{2}dx_{ij}$$

By algebraically manipulating the elasticity term of Equation (19), the following expression is obtained,

(20) $$V_{e_{ij}}=\frac{1}{2}\;z_{j}^{T}\;K_{ij}\;z_{j}$$

where $K_{ij}$ is the stiffness matrix of the *j* element of the $i^{th}$ link which is calculated as,

(21) $$K_{ij}=\frac{E_{ij}\;I_{i}}{l_{ij}^{3}}\;\left[\begin{array}{ccccc} 0_{n_{r}\times n_{r}} & 0_{1\times n_{r}} & 0_{1\times n_{r}} & 0_{1\times n_{r}} & 0_{1\times n_{r}}\\ 0_{n_{r}\times 1} & 12 & 6l_{ij} & -12 & 6l_{ij}\\ 0_{n_{r}\times 1} & 6l_{ij} & 4l_{ij}^{2} & -6l_{ij} & 2l_{ij}^{2}\\ 0_{n_{r}\times 1} & -12 & -6l_{ij} & 12 & -6l_{ij}\\ 0_{n_{r}\times 1} & 6l_{ij} & 2l_{ij}^{2} & -6l_{ij} & 4l_{ij}^{2} \end{array}\right]$$

and $0_{n_{r}\times n_{r}}$ is a $n_{r}\times n_{r}$ dimension null matrix, $n_{r}$ is the number of rigid variables of the system, $E_{ij}$ is the Young’s modulus of the element and $I_{i}$ is the moment of inertia of the link *i*.

In order to determine the deflection of each link, the generalised inertia and the stiffness submatrices concerning the flexible variables of each link ($M_{i_{f}}$ and $K_{i_{f}}$ respectively) have to be defined. As seen before, the generalised inertia and stiffness of each link is obtained by adding the inertia $M_{ij}$ and the stiffness $K_{ij}$ of all the elements that compose each link. From these matrices, the submatrices associated to the flexible variables $q_{f}$ are selected.

As length $l_{ij}$ and the Young’s modulus $E_{ij}$ of each element are the same for all elements of a given link, the structure of the obtained matrices is the similar, as it can be seen in the following example for a three element link,

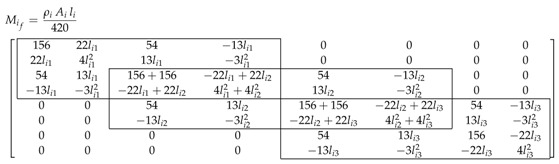
(22)
and

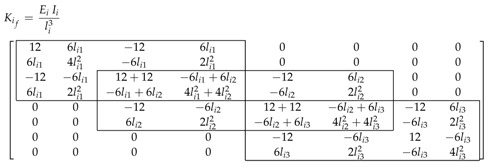
(23)
where $l_{i}=l_{i1}=l_{i2}=l_{i3}$ is the length of each element of link *i*, $\rho_{i}=\rho_{i1}=\rho_{i2}=\rho_{i3}$ is the mass density, $A_{i}=A_{i1}=A_{i2}=A_{i3}$ the cross-section area *i* and $E_{i}=E_{i1}=E_{i2}=E_{i3}$ the Young modulus of link *i*.

Once the generalised inertia submatrix $M_{i_{f}}$ and the stiffness submatrix $K_{i_{f}}$ of each limb are obtained, the boundary conditions of each flexible link have to be defined, this is, the deflection, the slope, the shear force and/or the bending moment in both ends of each link (x=0 and $x=l_{T_{i}}$). Depending on the link’s configuration, different Boundary Conditions (BC) are defined. As an example, when the link is working as a simply supported beam, both displacement and bending moment are set to zero at both ends. In a clamped configuration, on the contrary, only displacement and the slope are set to zero at the clamped node.

### 2.3. Modal Analysis of the Flexible Links

Once calculated the inertia and stiffness submatrices, the modal analysis procedure can be applied. For that purpose, a reduced set of equations of motion is required. In these equations the damping and the applied load are not taken into account,

(24) $${\left(M_{i_{f}}\right)}_{BC}\;\ddot{q}+{\left(K_{i_{f}}\right)}_{BC}\;q=0$$

To solve Equation (24), a harmonic solution as shown in Equation (25) is proposed.

(25) $$q_{f}=X\;sin\left(\omega \;t\right)$$

where ω is the natural frequency vector.

The harmonic solution is not only required for the numerical solution of the problem, but also for the physical interpretation of the equation. This way, the harmonic solution defines the way in which all the degrees of freedom of the link deflect and their relationship. Therefore, for a given mode, the structural configuration does not change during motion, only its amplitude, being possible to relate the effects of flexibility on each DOF if one of them is measured. This concept is the key to develop the proposed virtual sensor, as stated in the beginning of Section 2.

By substituting Equation (25) into Equation (24), the following equation is obtained.

(26) $$-\omega^{2}\;{\left(M_{i_{f}}\right)}_{BC}\;X\;sin\left(\omega \;t\right)+{\left(K_{i_{f}}\right)}_{BC}\;X\;sin\left(\omega \;t\right)=0$$

which after simplifying becomes

(27) $$\left({\left(K_{i_{f}}\right)}_{BC}-\omega^{2}\;{\left(M_{i_{f}}\right)}_{BC}\right)\;X=0$$

Equation (27) is called the eigenequation, which is a set of homogeneous algebraic equations for the components of the eigenvector, which forms the basis for the eigenvalue problem. The basic structure of the eigenvalue problem is defined as,

(28) (A−λI)X=0

where A is a square matrix, λ are the eigenvalues, I is an identity matrix of the same size as A and X is the eigenvector.

In the structural analysis of the limb of a robot, as in any other mechanical structure, the use of the generalised inertia matrix ${\left(M_{i_{f}}\right)}_{BC}$ and the stiffness matrix ${\left(K_{i_{f}}\right)}_{BC}$ gives a physical meaning to the eigenequation solution, being the obtained eigenvalues the natural frequencies of the system ($\lambda =\omega^{2}$) and the obtained eigenvector X the mode shapes of the system.

Solving Equation (27), two mathematically possible solutions can be obtained:

1. If $det\left({\left(K_{i_{f}}\right)}_{BC}-\omega^{2}\;{\left(M_{i_{f}}\right)}_{BC}\right)\neq 0$, then the solution is
  (29) X=0
  This is a trivial solution, which does not provide any valuable information from a physical point of view, since it represents the case of no motion.
2. If X≠0, the result of the eigenproblem is reduced to solve Equation (30).
  (30) $$det\left({\left(K_{i_{f}}\right)}_{BC}-\omega^{2}\;{\left(M_{i_{f}}\right)}_{BC}\right)=0$$
  The determinant defined in Equation (30) can only be zero at a set of discrete eigenvalues $\omega_{i}^{2}$. Furthermore, there is an eigenvector X which satisfies the equation Equation (27). Therefore, Equation (27) can be rewritten as:
  (31) $$\left({\left(K_{i_{f}}\right)}_{BC}-\omega_{i}^{2}\;{\left(M_{i_{f}}\right)}_{BC}\right)\;X_{i}=0\;i=1,2,3\ldots ,n_{DOF}$$
  where the number of eigenvalues and eigenvectors $n_{DOF}$ is equal to the number of DOF of the discretised limb has. Moreover, the *i*th eigenvalue $\omega_{i}^{2}$ is related to the *i*th natural frequency, $f_{i}$, by
  (32) $$f_{i}=\frac{\omega_{i}}{2\pi }$$

Hence, by solving Equation (31), the eigenvector $X_{i}$ can be calculated, which, in fact, represents the relationship between the flexible DOF of the link as it has been shown in Equation (4). As stated in the introduction of Section 2, in the proposed procedure, this calculation can be carried out off-line, as the inertia and stiffness submatrices do not depend on the particular position of the robot. This way, once calculated the eigenvalues for a given mode, if one of the flexible DOF is measured, the rest can be estimated.

Note that this approach is based on a simplified flexible model based on the discretization of the flexible links. However, its simple structure makes it computationally efficient, and, as it will be proved in the next section, it allows to estimate the deformation of flexible links with accuracy.

## 3. Case of Study: Delta Robot

In the following section, the developed virtual sensor will be implemented into a flexible-link parallel robot in order to validate it. For that purpose, the procedure detailed in the previous section will be followed.

### 3.1. The Delta Robot

The Delta robot is one of the most popular three DOF parallel robot, being widely used in industry. Its lightweight structure provides dynamic capabilities for quick motions, being mainly used for pick and place applications, in which several models can achieve up to 300 picks per minute.

**Figure 4:**
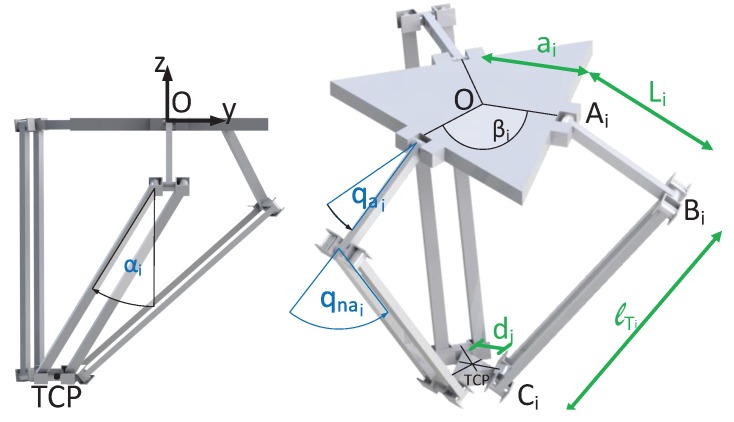
Robot schematic with the most important parameters and variables.

The robot is composed of 3 arms (i=1,2,3) distributed uniformly ($\beta_{1}=0$ rad, $\beta_{2}=2\;\pi /3$ rad, $\beta_{3}=4\;\pi /3$ rad) that connect the fixed based with the mobile platform. Each of these arms is composed by two limbs: the upper one is connected to the fixed base by actuated rotational joints, this is, they are connected to the actuators ($q_{a_{i}}$ for i=1,2,3); the lower ones, on the other hand, are composed by an articulated parallelogram which allows passive rotations in two directions ($q_{na_{i}}$ and $\alpha_{i}$ for i=1,2,3), as it can be seen in Figure 4. This configuration limits the motion of the mobile platform, and therefore the motion of the TCP, to a pure three dimensional translational motion with no rotations.

For this study case, the main parameters have been obtained from a commercial Omron Mini Delta CR-UGD4MINI-NR robot (Omron, Kioto, Japan), whose model has also been implemented in ADAMS Multibody Software (2014.0.1, MSC Software, Newport Beach, CA, USA). These parameters are summarised in Table 1.

In order to increase the effect of deformations, the links which compose the lower limbs have been replaced by AW5083/H111 aluminium platens (IMH, Elgoibar, Spain). These platens have 0.003 m thickness and a width of 0.015 m, and the used aluminium presents a Young’s modulus of $E_{i}=71$ GPa and a mass density of $\rho_{i}=2740$ kg/m^3^. The geometry has been selected in order to limit the deformation to the direction of the $z_{i}$ axis of each link, and analyse the validity of the proposed approach. All these proprieties are summarised in Table 2.

### 3.2. Numeric Implementation of the Kinematic Virtual Sensor Applied to the Delta Robot

**Figure 5:**
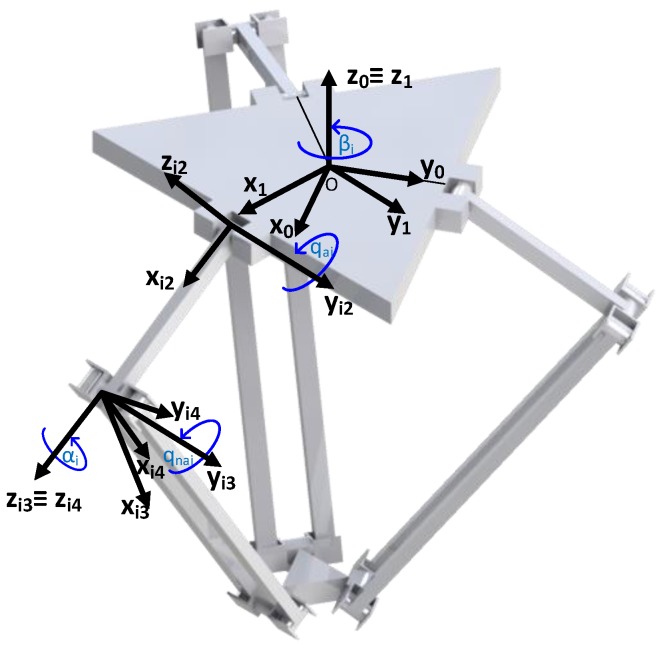
The Delta’s coordinate reference systems representation.

Based on the procedure detailed in Section 2 the first step to define the virtual sensor for the estimation of the links deflection is to define the transformation matrices between the inertial system $OX_{0}Y_{0}Z_{0}$, and the body-fixed system $O_{i4}X_{i4}Y_{i4}Z_{i4}$ , i=1,2,3 (Figure 5).

In order to place these reference systems, a systematic procedure is used. The most used procedure in parallel manipulators is the Equivalent Rigid Link System (ERLS) description based on the notation of Khalil and Kleinfinger [40] which is an adaptation of the well-known Denavit-Hartenberg notation [41] for closed-loop robots. However, this procedure has been adapted to provide a more systematic approach in flexible robots. This way, the *x* axis is always set along the length of the link, the *z* axis along the deformation plane of the link and the *y* axis perpendicular to the xz plane. Hence, the transformation matrix $T_{0}^{i}$ for each kinematic loop of the Delta Robot is defined as,

(33) $$T_{0}^{i}=T_{0}^{i1}\;T_{i1}^{i2}\;T_{i2}^{i3}\;T_{i3}^{i4}$$

where

(34) $$\begin{array}{cc} T_{i0}^{i1} & =\left[\begin{array}{ccc} cos(\beta_{i}) & -sin(\beta_{i}) & 0\\ sin(\beta_{i}) & cos(\beta_{i}) & 0\\ 0 & 0 & 1 \end{array}\right],\;T_{i1}^{i2}=\left[\begin{array}{ccc} cos(q_{a_{i}}) & 0 & sin(q_{a_{i}})\\ 0 & 1 & 0\\ -sin(q_{a_{i}}) & 0 & cos(q_{a_{i}}) \end{array}\right]\\ T_{i2}^{i3} & =\left[\begin{array}{ccc} cos(q_{na_{i}}) & 0 & sin(q_{na_{i}})\\ 0 & 1 & 0\\ -sin(q_{na_{i}}) & 0 & cos(q_{na_{i}}) \end{array}\right],\;T_{i3}^{i4}=\left[\begin{array}{ccc} cos(\alpha_{i}) & -sin(\alpha_{i}) & 0\\ sin(\alpha_{i}) & cos(\alpha_{i}) & 0\\ 0 & 0 & 1 \end{array}\right]\\ for\;i=1,2,3 & \end{array}$$

In order to simplify the mathematical model, the articulated parallelogram has been reduced to a single equivalent link. This assumption has proven to be valid after analysing the dynamic behaviour of the articulated parallelogram link in simulations, in which it was observed that both elements of the parallelogram present the same dynamic behaviour at lower natural frequencies.

The Finite Element Method is then applied to model the deformation of the links. This approach requires to define the number of elements considered per link. Tsujisawa suggested in [42] that for robot manipulators a relatively small number of modes (two or three) are usually enough to represent the dynamics of flexible links. Furthermore, according to Przemieniecki’s work [43], when an *m*-element FEM model is used, the system’s first *m* vibration modes can be obtained with acceptable accuracy. Hence, for the developed virtual sensor a three element FEM model per flexible link will be used.

Once the number of elements per link is defined, the generalised inertia matrix and the stiffness matrix of each flexible link has to be calculated. As explained in Section 2.2, for the developed approach, just the submatrix concerning the flexible variables, $M_{i_{f}}$ and $K_{i_{f}}$ respectively, are required. These submatrices have the main advantage that are constant, since they are calculated in terms of the mechanical parameters, which are invariable in all the elements of a given link.

Hence, for a three-element FEM model platens,

(35) $$M_{1_{f}}=M_{2_{f}}=M_{3_{f}}=\frac{\rho_{i}\;A_{i}\;l}{420}\;\left[\begin{array}{cccccccc} 156 & 22l & 54 & -13l & 0 & 0 & 0 & 0\\ 22l & 4l^{2} & 13l & -3l^{2} & 0 & 0 & 0 & 0\\ 54 & 13l & 312 & 0 & 54 & -13l & 0 & 0\\ -13l & -3l^{2} & 0 & 8l^{2} & 13l & -3l^{2} & 0 & 0\\ 0 & 0 & 54 & 13l & 312 & 0 & 54 & -13l\\ 0 & 0 & -13l & -3l^{2} & 0 & 8l^{2} & 13l & -3l^{2}\\ 0 & 0 & 0 & 0 & 54 & 13l & 156 & -22l\\ 0 & 0 & 0 & 0 & -13l & -3l^{2} & -22l & 4l^{2} \end{array}\right]$$

and the stiffness submatrix as

(36) $$K_{1_{f}}=K_{2_{f}}=K_{3_{f}}=\frac{E_{i}\;I_{i}}{l}\;\left[\begin{array}{cccccccc} 12 & 6l & -12 & 6l & 0 & 0 & 0 & 0\\ 6l & 4l^{2} & -6l & 2l^{2} & 0 & 0 & 0 & 0\\ -12 & -6l & 24 & 0 & -12 & 6l & 0 & 0\\ 6l & 2l^{2} & 0 & 8l^{2} & -6l & 2l^{2} & 0 & 0\\ 0 & 0 & -12 & -6l & 24 & 0 & -12 & 6l\\ 0 & 0 & 6l & 2l^{2} & 0 & 8l^{2} & -6l & 2l^{2}\\ 0 & 0 & 0 & 0 & -12 & -6l & 12 & -6l\\ 0 & 0 & 0 & 0 & 6l & 2l^{2} & -6l & 4l^{2} \end{array}\right]$$

where $l=l_{i}=\frac{l_{T_{i}}}{3}$.

Finally, the boundary conditions (BC) to each flexible link must be defined. It is a general mathematical principle that the number of BC necessary to calculate the solution to a differential equation matches the order of the differential equation. The used Euler-Bernoulli beam model is a fourth-order equation, so each flexible link requires four boundary conditions to be solved.

According to the literature, different types of boundary conditions can be used. The BC associated to a Pinned-Pinned beam model, for instance, has been used for trajectory control of the TCP of the robot [44] due to its simplicity when implementing the position problem of the TCP. The boundary conditions associated to Free-Free beams have also been used with rigid motion variables [45]. Finally, other authors, have considered the flexible link as a Clamped Beam, demonstrating that their use simplifies the measurement of joint variables and the calculation of required torques [46,47].

According to the experimental verifications reported in [26,48], if the beam to hub inertia ratio is very small, (at least ten times smaller) the Clamped Beam boundary condition set yields better results compared to Pinned-Pinned boundary condition set. Hence, for the developed virtual sensor, Clamped Beam boundary conditions will be used:

- The base of the link does not experience any deflection: $z_{i}\left(0\right)=0$.
- The link at the base has no deformation, so that the derivative of the deflection function is zero at that point: $\frac{\partial z_{i}\left(0\right)}{\partial x}=0$.
- There is no bending moment at the end of the link: $\frac{\partial^{2}z_{i}\left(l_{T_{i}}\right)}{\partial x^{2}}=0$.
- There is no shearing force acting at the end of the link: $\frac{\partial^{3}z_{i}\left(l_{T_{i}}\right)}{\partial x^{3}}=0$.

If these boundary conditions are introduced in the developed model,

- If $z_{i}\left(0\right)=0$ then $u_{i1}=0$.
- If $\frac{\partial z_{i}\left(0\right)}{\partial x}=0$ then $u_{i2}=0$.

Therefore, the first two lines and columns of the $M_{i_{f}}$ and $K_{i_{f}}$ can be erased, defining the new generalized inertia submatrix as,

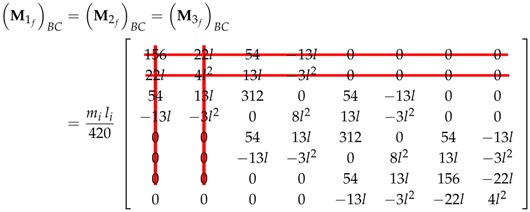
(37)
and the stiffness submatrix as

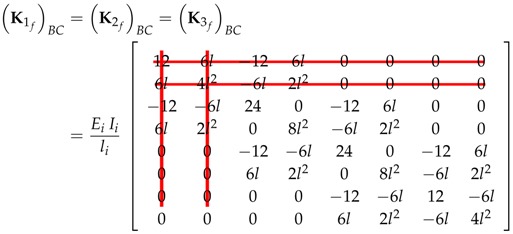
(38)

Once inertia and stiffness submatrices have been defined, based on Equation (31) the natural frequencies $\omega_{i}$ and the eigenvector $X_{i}$ can be obtained,

(39) $$\left[X_{i},\omega_{i}\right]=eig\left({\left(K_{i_{f}}\right)}_{BC},{\left(M_{i_{f}}\right)}_{BC}\right)$$

As explained in Section 2.3, the obtained eigenvector $X_{i}$ defines the relationship between the displacement and the slope of the nodes of the link. The same obtained eigenvector, not only holds for the position problem relationship, but also for the velocity and acceleration problems relationship, which makes the base of the proposed virtual sensor.

**Figure 6:**
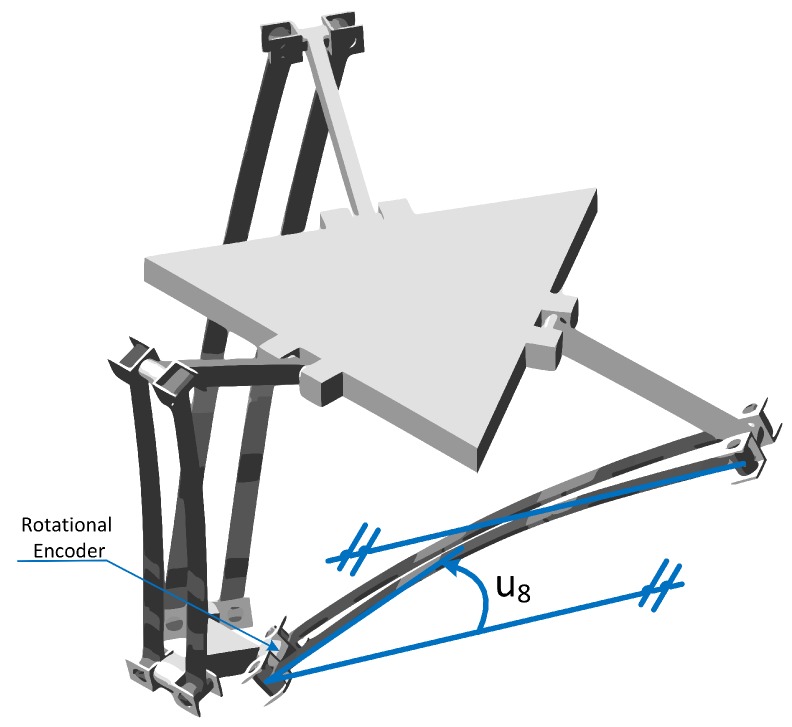
Rotational encoder position and the measured angle representation.

Hence, Equation (39) allows to calculate the modal displacement or slope of all the nodes of the *i*th link using the measurement of a single displacement or slope. Moreover, the measurement of the slope of the flexible link’s tip ($u_{i8}$) is quite straightforward in parallel robots (Figure 6) by the use of precision encoders. Hence, using a single sensor, the deformation ($q_{if}$), velocities (${\dot{q}}_{if}$) and acceleration (${\ddot{q}}_{if}$) of the flexible link’s DOF can be estimated easily using the virtual sensor Equation (39).

### 3.3. Simulation Setup

**Figure 7:**
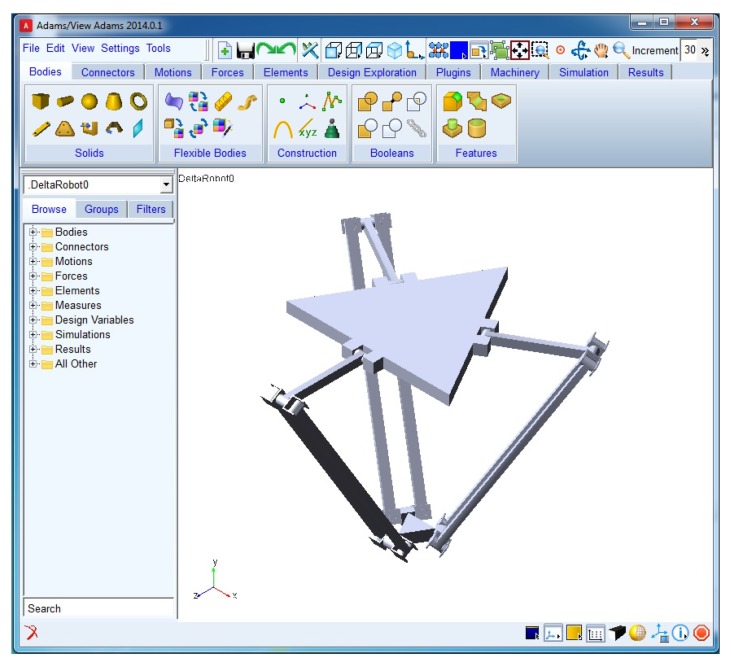
Image of the simulated Flexible Delta Robot in ADAMS.

The virtual sensor is validated next. For that purpose, the Delta robot detailed in Section 3.1 has been modelled in ADAMS Multibody Software (2014.0.1, MSC Software, Newport Beach, CA, USA), (Figure 7). Flexibility parameters (Table 2) have also been introduced into the software. 

**Figure 8:**
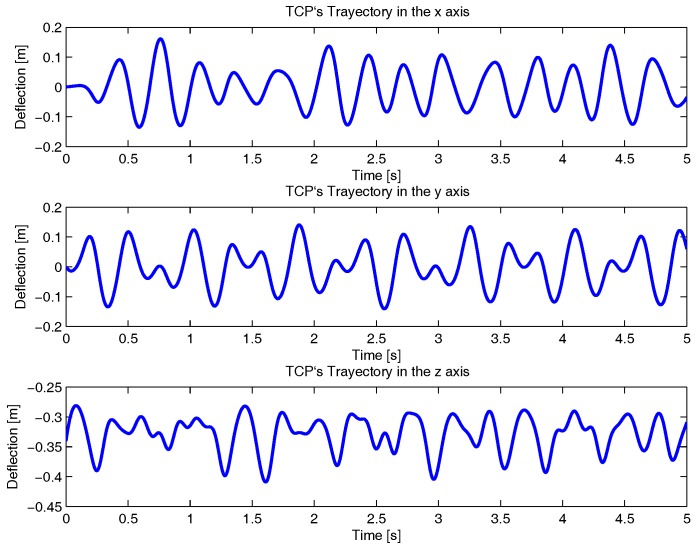
The reference trajectory of the TCP for the defined movement of the motors.

In order to analyse the flexible behaviour of the links of the Delta robot and validate the proposed approach, the robot is excited in a wide range of frequencies within the operational bandwidth of the robot. Hence, a sinusoidal motion of different frequencies has been applied in the actuated joints $q_{a_{i}}$,

(40) $$\tau_{1}=0.5\sin\left(2\pi \;\omega_{\tau_{1}}\;t\right)\;\tau_{2}=0.5\sin\left(2\pi \;\omega_{\tau_{2}}\;t\right)\;\tau_{3}=0.5\sin\left(2\pi \;\omega_{\tau_{3}}\;t\right)$$

where $\omega_{\tau_{1}}=3$ rad/s, $\omega_{\tau_{2}}=0.75\cdot 3$ rad/s, $\omega_{\tau_{3}}=1.2\cdot 3$ rad/s and *t* is the time, which is defined between 0 and 5 s. With it, the obtained TCP movement if shown in Figure 8.

Three additional sensors will be considered in the robot. As stated in the previous section, the virtual sensor can determine the deformation of the link by measuring a single deformation slope, which in the case of rotary joints can be easily measured by the use of three encoders installed in the passive rotary joints of the mobile platform (Figure 6).

The validation of the proposed kinematic virtual sensor has been carried out considering two factors. First, the evaluation of the virtual sensor capability to estimate the deformation of the flexible links based on a single encoder measurement will be analysed. According to FEM definition, each of the flexible links is composed by a certain number of nodes, each of which have two DOF, the displacement and the slope deflection. Hence, in order to analyse the accuracy of the virtual sensor, the estimation of each DOF is compared with the measurements obtained from ADAMS Multibody Software. As explained in previous sections, the developed virtual sensor not only estimates the position and the orientation of the DOF, but also the speed and the acceleration of them. Due to it, the accuracy of the virtual sensor will be analysed in position, speed and acceleration.

**Figure 9:**
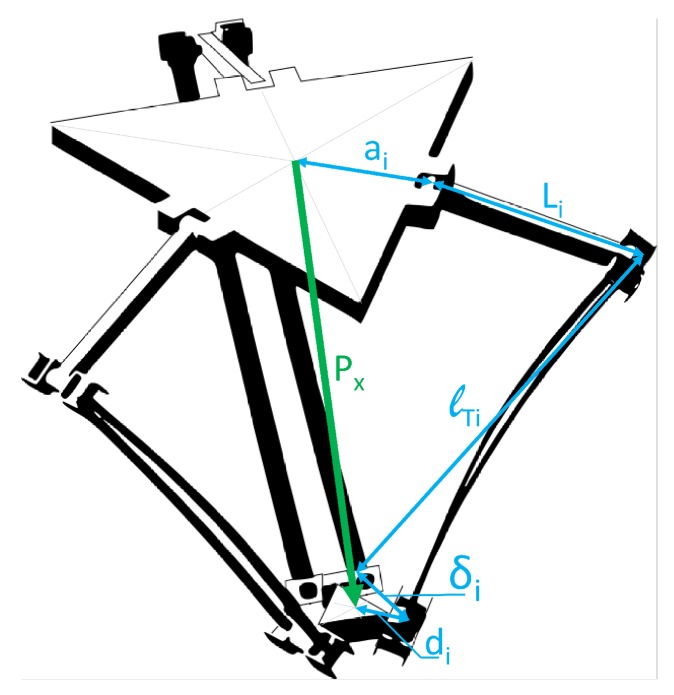
Graphical representation of the loop closure of a Delta Robot.

On the other hand, the performance of the virtual sensor will be evaluated for its application to the calculation of the Direct Kinematic Problem of flexible parallel robots. This model is used to estimate the motion of the TCP in terms of the joint sensors of the robot and the virtual sensors introduced to measure the flexibility. The kinematic model is derived from the loop closure equations of the mechanism, which relate the kinematic variables of the robot, such as the angle of the joints, the position of the TCP and the deformations due to flexibility [1]. For the particular case of the analysed Delta robot, these equations are (Figure 9),

(41) $$\Gamma_{i}\left(x,q\right)=a_{i}+L_{i}\left(q_{a}\right)+l_{T_{i}}\left(q_{a},q_{\mathrm{na}},\alpha_{i}\right)+\delta_{i}-d_{i}-p_{x}=0$$

where i=1,2,3 is the link identification and $\delta_{i}$ is the deflection distance in the flexible link end which respect to its analogous rigid link. This last variable is the one to be estimated by the virtual sensor.

Hence, the position of the TCP of the robot, $p_{x}$ is calculated as,

(42) $$\begin{array}{c} p_{x}=T_{i0}^{i1}\;\left[\begin{array}{c} a_{i}\\ 0\\ 0 \end{array}\right]+T_{i0}^{i1}\;T_{i1}^{i2}\;\left[\begin{array}{c} L_{i}\\ 0\\ 0 \end{array}\right]+T_{i0}^{i1}\;T_{i1}^{i2}\;T_{i2}^{i3}\;T_{i3}^{i4}\;\left[\begin{array}{c} l_{T_{i}}\\ 0\\ \delta_{i} \end{array}\right]-T_{i0}^{i1}\;\left[\begin{array}{c} d_{i}\\ 0\\ 0 \end{array}\right]\\ for\;\;i=1,2,3 \end{array}$$

Next, the results obtained from both analysis will be discussed.

## 4. Results and Discussion

**Figure 10:**
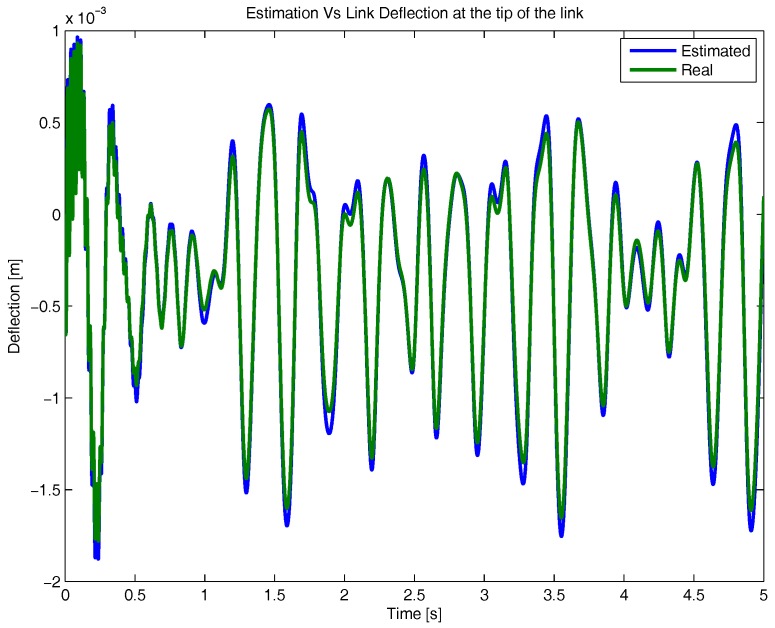
Virtual Sensor link deflection estimation compared with the real deflection at the tip of the link obtained with ADAMS Multibody Software.

First, the accuracy of the virtual sensor to estimate the deformation of each flexible link will be analysed. For that purpose, the deflection estimation of a single flexible link tip provided by the virtual sensor has been compared with the data provided by ADAMS Multibody software. Results are shown in Figure 10, where it has been assumed that the first natural frequency amplitude was dominant in the estimator. 

As it can be seen in Figure 10, the estimation is accurate. Data shows that the virtual sensor is able to estimate the deflection of the tip with a maximum error of less than 0.1 mm, which is less than the 2% of the maximum deflection amplitude.

**Figure 11:**
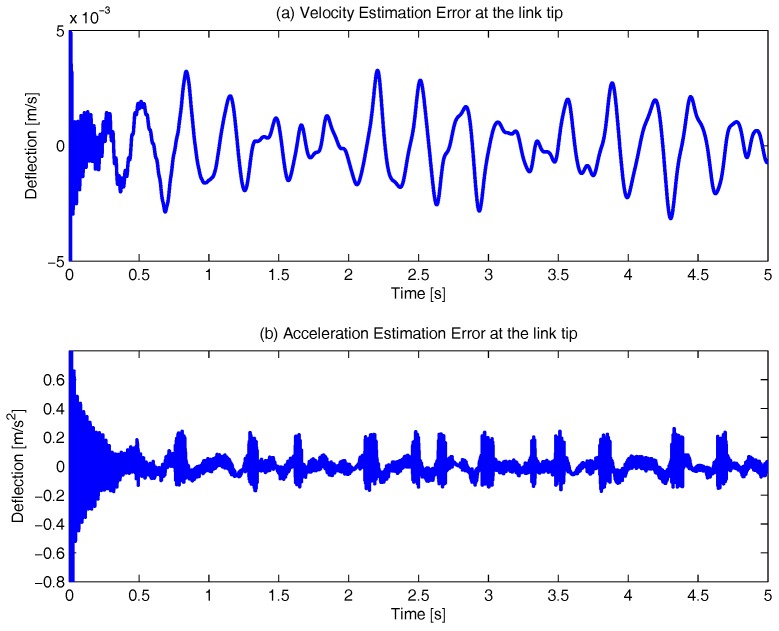
Virtual Sensor link deflection speed and acceleration error.

Furthermore, the proposed virtual sensor does not only establish the relationship between the displacements and the orientations of each link’s nodes, but also their speed and acceleration. Hence, following the same procedure, the speed error of the flexible link tip, Figure 11a, and its acceleration error, Figure 11b, can be obtained. 

Figure 11a shows that the maximum error is $3\times 10^{-3}$ m/s, which means a speed error of less than the 6% of the amplitude of the signal. In the same way, in Figure 11b the acceleration error is plotted, in which the maximum value is 0.2 m/s^2^, a relative error of less than the 13% of its amplitude.

**Figure 12:**
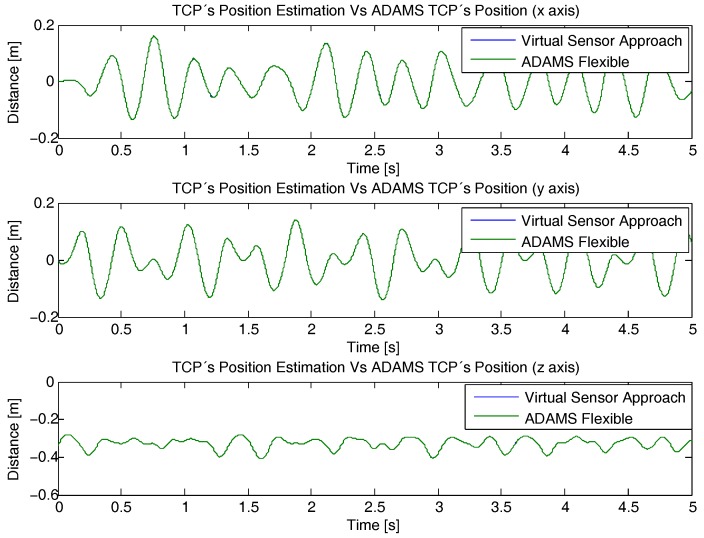
Virtual Sensor TCP deflection estimation compared with the TCP’s deflection obtained with ADAMS Multibody Software.

Next, the deflection data calculated by the virtual sensor is used to compute the Direct Kinematic Problem (Equation (41)). For that purpose, the estimation error of the TCP considering the virtual sensor is compared with the TCP trajectory shown in Figure 8, which was obtained from the flexible ADAMS Multibody Software model, as it is shown in Figure 12.

**Figure 13:**
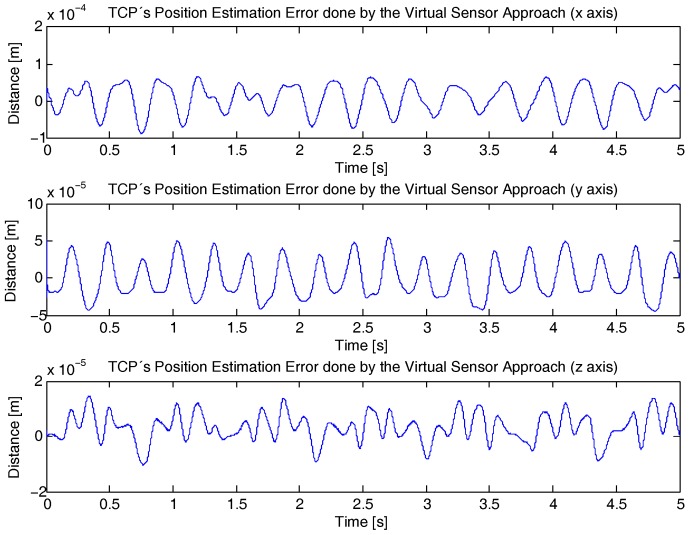
Virtual Sensor TCP deflection estimation error compared with the TCP’s deflection obtained with ADAMS Multibody Software.

This way, in Figure 13 the *x*, *y* and *z* component of the TCP positioning error of the developed estimator is shown. As it can be seen, the estimation error of the developed approach rises up to $10^{-4}$ m for the *x* axis, $5\times 10^{-5}$ m for the *y* axis and $1.5\times 10^{-5}$ m for the *z* axis, which means an error of less than the 0.03%, 0.02%, 0.007% of the applied movement, respectively.

Hence, the aforementioned results demonstrate that the developed approach presents great potential for the estimation of deflections in flexible robots. However, it is important to note that in order to obtain accurate estimation results the proposed virtual sensor needs to consider the bandwidth of the motors and ensure that the range of the estimator is inside it. Furthermore, an analysis of the resolution of the encoder needs to be carried out so that the precision requirements of the estimation are satisfied. Finally, although implementation issues are not the focus of this work, it is important to analyze the computational cost requirements of the approach when applied to a particular robot, and select an appropriate hardware to ensure that the estimation is carried out within the time constraints of the control loop.

## 5. Conclusions

Parallel robots are widely used in industry for high dynamic requirement tasks that imply high speed. In order to achieve these capabilities, the structure of the robot is usually lightweight, so that small deflections can arise due to high speed motions. Modern controllers can compensate the deformations caused by the elastic deformation of the links. However, the measurement of small deformations is a challenging tasks that usually require extra sensors and complex models.

In this work, a novel virtual sensor for the estimation of the deformation of flexible links in parallel robots is presented. The developed approach is based on the modal analysis of the flexible link, which is modelled based on Finite Element Method approach and the Euler Bernouilli Beam theory. This approach is focused on control applications, providing a simple yet computationally effective approach that provides accurate estimations in comparison with other approaches.

The proposed virtual sensor has been validated in a flexible Delta Robot which has been modelled on ADAMS Multibody Software. The deformation data obtained in ADAMS was compared with the one estimated by the virtual sensor, resulting in a maximum error of less than 2% for the deformation estimation and 6% and 13% for the speed and acceleration estimation, respectively.

In addition, using a similar procedure, the virtual sensor estimation was used to estimate the Direct Kinematic Problem of the flexible Delta Robot. This data was compared with the flexible Delta model developed in ADAMS Multibody Software, resulting in a TCP error estimation of less than 0.03% (0.03%% for the *x* axis, 0.02% for the *y* axis and 0.007% for the *z* axis).

## Figures and Tables

**Table 1:** Parameters of Omron Mini Delta CR-UGD4MINI-NR.

	Fixed Base	Upper Link $L_{i}$	Lower Link $l_{T_{i}}$	Mobile Platform
Length (m)	$|a_{i}|=0.100$	0.150	0.400	$|d_{i}|=0.040$
Mass (kg)		0.0365	0.1319	0.1278
		$I_{L_{i_{xx}}}=2.2781\times 10^{-6}$	$I_{l_{i_{xx}}}=2.0023\times 10^{-6}$	$I_{p_{xx}}=4.6225\times 10^{-5}$
Inertia (kg m^2^)		$I_{L_{i_{yy}}}=8.7001\times 10^{-5}$	$I_{l_{i_{yy}}}=0.0010$	$I_{p_{yy}}=4.6225\times 10^{-5}$
		$I_{L_{i_{zz}}}=8.6422\times 10^{-5}$	$I_{l_{i_{zz}}}=0.0010$	$I_{p_{zz}}=9.1472\times 10^{-5}$

**Table 2:** Mechanical properties of the aluminium platens.

Length	Width	Thickness	Young’s Modulus	Mass Density
0.400 m	0.015 m	0.003 m	71 GPa	2740 kg/m^3^

## Abbreviations

The following abbreviations are used in this manuscript:

PKR	Parallel Kinematic Robot
TCP	Tool Centre Point
DOF	Degree of Freedom
AMM	Assumed Modes Method
FEM	Finite Element Method
ERLS	Equivalent Rigid Link System
BC	Boundary Condition
DKP	Direct Kinematic Problem
